# A point-of-care assay for genotyping women prior to ovarian stimulation with recombinant or human menopausal gonadotropins in assisted reproduction

**DOI:** 10.3389/fendo.2026.1697345

**Published:** 2026-01-28

**Authors:** Mathilda Nilsson, Ida Hjelmér, Alexandra Prahl, Hannah Nenonen, Yvonne Lundberg Giwercman

**Affiliations:** Department of Translational Medicine, Molecular Genetic Reproductive Medicine, Clinical Research Centre, Lund University, Malmö, Sweden

**Keywords:** FSHR, IVF, LAMP, ovarian stimulation, precision medicine, polymorphism

## Abstract

**Objective:**

Prior to *in vitro* fertilization (IVF), physicians can choose either recombinant or urine-derived follicle-stimulating hormone (FSH) for ovarian stimulation. The common polymorphism N680S (rs6166) in the follicle-stimulating hormone receptor (FSHR) has been linked to individual variability in ovarian response to FSH stimulation and IVF outcomes, including live birth rates, highlighting its potential value in optimizing stimulation protocols. However, classical genotyping is laborious, often requiring blood as starting material and specialized laboratories for analysis. The aim of the study was to develop a system for rapid and easy-to-use genotyping of the FHSR N680S variant.

**Methods:**

The assay constituted an allele-specific peptide nucleic acid-mediated loop-mediated isothermal amplification (AS PNA-mediated LAMP) assay with a colorimetric detection system. The analytical performance was analyzed with naked-eye detection and verified with fluorescence amplification. Clinical validation was assessed on 50 patients visiting an IVF-clinic in whom the variant was confirmed with Sanger sequencing.

**Results:**

Ninety-one out of 100 samples were genotyped correctly, demonstrating 91% overall accuracy. Clinical sensitivity reached 86.8 [95% Cl 76.4-93.8%], while specificity was 100% [95% Cl 89.1-100%]. The test was performed by trained laboratory staff in less than one hour.

**Conclusion:**

The LAMP assay provides a rapid and user-friendly genotyping of the FSHR N680S, which makes it a valuable tool in point-of-care settings, where it may help guide treatment options prior to IVF.

## 1 Introduction

The gonadotropins follicle-stimulating hormone (FSH) and luteinizing hormone (LH) regulate the function of both ovaries and testicles (1). In women, FSH released from the pituitary gland binds to the FSH receptor (FSHR) localized in the granulosa cell membrane and stimulates antral follicular growth before ovulation (2). In men, FSH stimulates spermatogenesis by binding to the FSHR on Sertoli cells (3).

It has long been known that the adenine to guanine single-nucleotide polymorphism (SNP), which results in the asparagine (N) to serine (S) substitution in codon 680 (FSHR N680S, rs6166) is a crucial regulator of receptor sensitivity. Women carrying the homozygous S variant in general have higher endogenous serum FSH concentrations, indicating lower receptor sensitivity than those with N in the same position (4, 5). Often, these women also present with reduced ovarian response to stimulation when undergoing ovarian hormone stimulation and therefore require higher doses of FSH than N-carriers do (5, 6).

There are two types of FSH used for ovarian stimulation – recombinant (rFSH) and urine-derived (hMG), respectively. Higher live birth rates have been reported in hMG treated women carrying S alleles, whilst homozygous NN-carriers produced a higher number of mature oocytes if treated with rFSH (7). This was consistent with the results from a previous smaller study showing that egg donors homozygous for the SS variant produced more mature oocytes if they were treated with hMG, as compared to when the same women were administered rFSH (8). Regarding the homozygous NN variant, several clinical studies on women undergoing *in vitro* fertilization (IVF) have shown that carriers of this variant require lower total dose of exogenous FSH for ovulation, but still develop more oocytes when stimulated with rFSH than those with NS or SS in the same position (5, 7, 9, 10). Genotyping prior to hormonal treatment may therefore be advantageous for predicting IVF outcomes and for optimizing ovarian stimulation protocols, potentially leading to more effective IVF treatments with fewer cycles required per live birth, thereby lowering associated health-care costs and the physical and emotional burden on patients.

Implementation of genotyping in routine IVF depends on the availability of a simple, robust method that fits within established workflow. The current golden standard Sanger sequencing is time-consuming and costly in terms of equipment and laboratory resources required. Similarly, other commonly used genotyping systems, such as next-generation sequencing (11), allele-specific polymerase chain reaction (as-PCR) (12), competitive amplification of differentially melting amplicons (CADMA) (13), and TaqMan assay (14), depend on a PCR amplification of target DNA. Consequently, the need for accessory equipment such as thermal cyclers and laboratory skills limits the use of these methods in unspecialized facilities. In addition, the mentioned techniques typically rely on DNA obtained from venous blood samples, whereas non-invasive sampling methods, such as buccal swabs, may be better suited for routine IVF practice.

Loop-mediated isothermal amplification (LAMP) is an attractive alternative (15). In contrast to PCR, it utilizes four to six primers that specifically recognize regions on the target DNA and a DNA polymerase with strand displacement ability (16). Beyond high specificity, some studies have demonstrated the potential of using crudely processed blood and buccal swab samples (17, 18), rather than relying on DNA extracted from blood samples. By shortening turnaround time and minimizing hands-on work, this method addresses one of the main challenges for implementing genotyping in routine clinical care. Furthermore, as LAMP assay is performed under constant temperature, no thermal cycler is required, which reduces costs and enables application also in resource-limited environments or hospitals in remote areas.

Loop-mediated isothermal reactions can be carried out with fluorescent probe detection (19), nanoparticles (20), and even visualization by adding colorimetric indicators (21). In a colorimetric detection system, the change in color allows naked-eye discrimination between positive and negative reactions, corresponding to different genotypes (22). As previous results have indicated that S-carriers would benefit from stimulation with hMG, while NN-carriers benefit from rFSH (7), a method that can distinguish between these groups is desired.

The present study aimed to develop a non-invasive test requiring only a buccal sample. The requirements was that the test should be usable under simple conditions and allow for visual, color-based readout of the result. Eventually, a rapid and easy-to-use method suitable for point-of-care settings was developed.

## 2 Materials and methods

### 2.1 Study design

The study was designed in two parts. First, the LAMP assay was developed and validated using DNA extracted from blood. The analytical sensitivity and specificity were analyzed to detect minimum target input, and the sample collection procedure was optimized. In the second, clinical phase, the LAMP assay was validated for accuracy and precision by analyzing 1) n=400 DNA samples extracted from blood and 2) n=100 buccal samples in a real-world setting. The results were compared to Sanger sequencing of DNA from blood samples from the same persons.

### 2.2 Subjects and sample preparations

Venous blood samples collected from 400 subjects attending the Reproductive Medicine Center (RMC), Malmö, Sweden during the period 2016 to 2020 (7) were used for method development and for the first part of the clinical assessment. DNA was extracted from blood samples using a QIamp DNA Blood mini kit (Qiagen N.V., Hilden, Germany) and processed as described in Hjelmér et al., 2025. Next, to assess the complete workflow and clinical validation, including sampling, venous blood and buccal samples, respectively, were collected from 50 subjects consecutively attending RMC, Malmö, Sweden, during the period December 2024-November 2025 (n=25 men; n=25 women). Buccal samples were collected one from each cheek (in total n=100) by the study participants themselves by swabbing the inside of their cheeks for 30 seconds. Swabs were placed in tubes and frozen in -20°C until analysis. DNA was extracted by incubation in NaOH for 10 minutes at 65°C.

Current study was approved by the Swedish Ethical Review Board (2016-467; 2023-0546502; 2024-01055-02;2024-06038-02; 2024-03115-02). All participants signed written informed consents.

### 2.3 Allele-specific PNA-mediated LAMP

**Figure 1:**
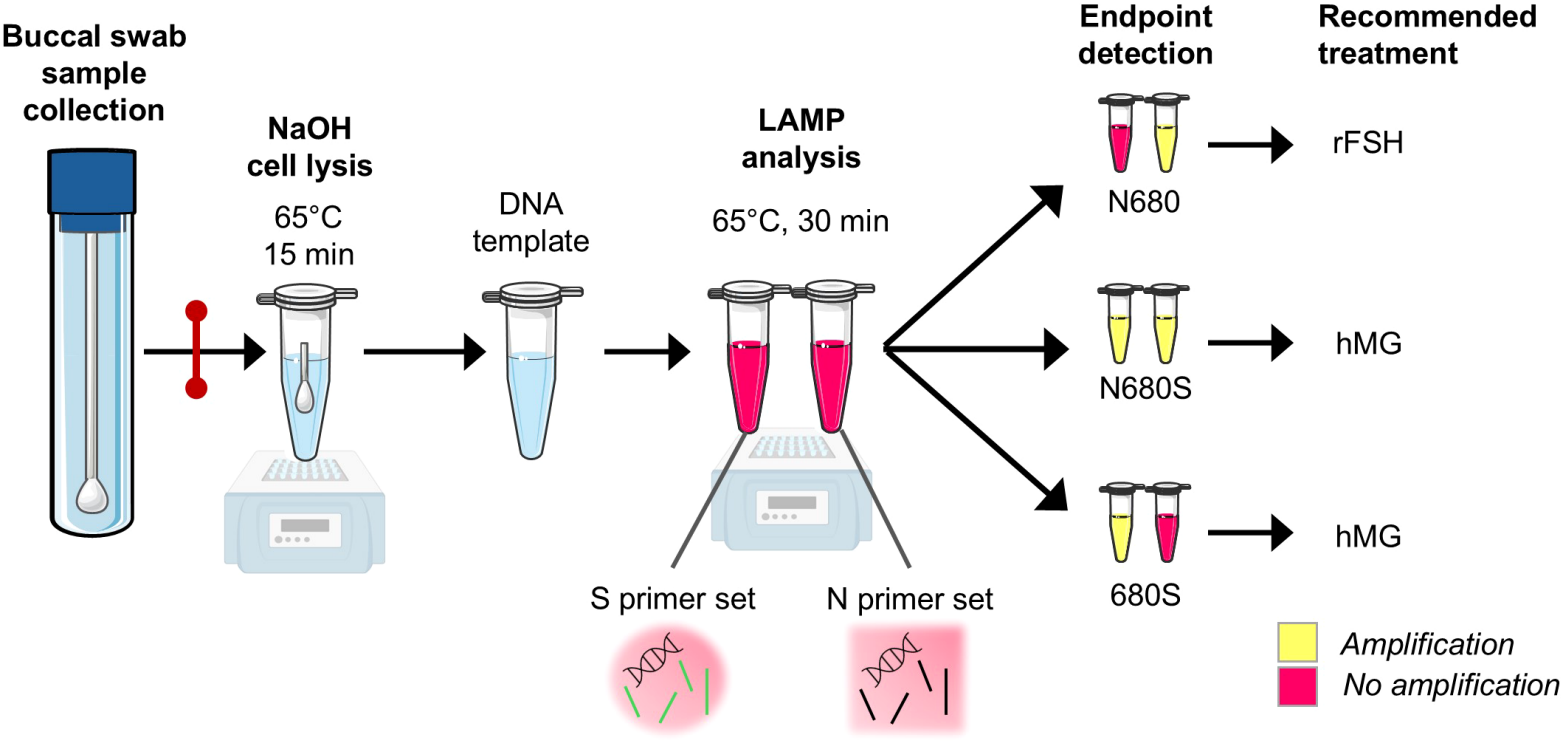
Schematic presentation of allele-specific PNA-mediated loop-mediated isothermal amplification workflow. Samples can be stored at 4°C or at -20°C for up to 2 weeks before proceeding to step 2, as indicated by the red line. LAMP loop-mediated isothermal amplification; S serine; N asparagine; FSH follicle-stimulating hormone; rFSH recombinant FSH, hMG human menopausal gonadotrophin.

The workflow of the developed allele-specific (AS) peptide nucleic acid (PNA)-mediated LAMP is illustrated in **Figure 1**. Buccal cells were collected with swabs. DNA was extracted by heated NaOH treatment and DNA amplification was achieved using a set of six primers designed to selectively bind one of the alleles in FSHR N680S. The two allele variants are referred to as the N-variant and the S-variant, respectively. Alle discrimination depends on the overlap between the SNP site and two of the primers. A single-base mismatch results in unstable hybridization and severely decrease amplification rate. The DNA amplification generates massive numbers of short-linked DNA amplicons to be used for detection. For complete genotyping, two parallel reactions (one S-specific and one N-specific) were run simultaneously.

A PNA clamp with a neutral backbone complementary to the alternative allele was designed to block the SNP site and added to serve as a blocking agent. Detection relied on visual interpretation of colorimetric pH-based indicators. The colorimetric assay was validated in a fluorescence-based assay using a DNA-binding dye.

### 2.4 Oligos

Two sets of LAMP primers for genotyping were designed using PrimerExplorer v5 online software (Primer-E Ltd., Plymouth, UK). To ensure sufficient discrimination between the N and S alleles, series of inner primers were designed and assessed for optimal conditions. All primers were purchased from Eurofins Genomic (Ebersberg, Germany).

In addition, two PNA clamps (Biomers GmbH, Ulm, Germany) specific to each allele of the FSHR N680S polymorphism were designed.

Two primers previously published (23) were utilized for PCR reaction and sequencing of the FSHR N680S variants.

### 2.5 Controls

Three pCMV6 XL5 vectors (OriGene Technologies GmbH, Herford, Germany) containing the N, the S allele or none of the alleles were transformed into electrocompetent E. coli (C3020K, New England Biolabs, Ipswich, Massachusetts, United States). The transformed bacteria were amplified and plasmids subsequently extracted (ExtractMe Plasmid DNA kit, Biolab Innovative Research Technologies [Blirt] S.A., DNA-Gdańsk).

The vector DNA sequences were verified by direct sequencing (ONT Lite Whole Plasmid Sequencing, Eurofins Genomics). The vector containing the S allele was used as positive control to ensure correct preparation and function of the genotyping reagent. After analysis, a color change indicated correct preparation. The empty vector was used as negative control to detect contaminations. These controls were included in all clinical performance analyses. Nuclease-free H_2_O (nF-H_2_O) was used as negative control in analytic performance analyses.

### 2.6 Genotyping by LAMP

To test the LAMP assay´s capability to genotype the FSHR N680S, two parallel reactions were run, containing a primer set specific for either the N-variant or the S-variant. Reactions were prepared in volumes of 25 µL containing WarmStart^®^ Colorimetric LAMP 2X Master Mix with UDG (New England Biolabs), nF-H_2_O, 0.2 µM – 1.6 µM FSHR N680S specific primers, PNA clamp [0.2 µM]) and 1 µL template DNA solution. The assay performance was evaluated in gradient temperatures and time intervals in a MyBlock Mini Dry Bath (Benchmark Scientific Inc, Sayreville, USA).

In the validation fluorescence-based assay, 1 µL SYBR Green Fluorescent DNA stain (Jena Bioscience, Jena, Germany) and 1 µL ROX Reference Dye (Jena Bioscience) was added. The samples were run in a Bio-Rad CFX thermocycler (Agilent Technologies, California, United States) for 50 minutes and monitored every 30 seconds in the SYBR channel. Genotypes were determined by analyzing differences in color changes, reaction times and amplification curves.

### 2.7 Analytic performance

Analytic performance was assessed to validate accuracy and reliability of the assay. The minimum input DNA concentration was evaluated by analytic sensitivity, analyzed by gradient dilution of the positive control template (10–10^9^ copies). Analytic specificity was assessed to measure how precise the assay could distinguish the target variant from the alternative variant by using mixed templates from 100% - 0% in total concentrations of 10^5^ copies of templates. The PNA was introduced to increase specificity. Genotyping was evaluated on samples with sequenced genotypes. To investigate how the assay could analyze crudely processed buccal swabs, the S-specific LAMP assay was assessed on swabs lysed in a gradient of NaOH concentrations (1, 25, 50, 100 mM), followed by heated (65° C) and room temperature (RT) lysis, respectively. All analyses were run in duplicates and repeated three times.

### 2.8 Genotype detection of clinical samples

The LAMP technique’s clinical performance regarding the FSHR N680S polymorphism was evaluated in two steps with the S-specific allele set as reference.

#### 2.8.1 Part 1

First, the 400 DNA samples extracted from venous blood samples were analyzed with the novel LAMP assay and results were compared with those derived by Sanger sequencing of the same samples.

#### 2.8.2 Part 2

A power calculation based on the observed 95% accuracy in step 1 proposed a sample size of 73 buccal swabs (80% power, 95% confidence interval, ± 5% margin of errors). Subsequently, 100 buccal samples were collected from patients attending fertility investigation (2024-2025). The laboratory staff at the clinic analyzed the samples blindly and the results were compared with DNA sequencing of matched blood samples.

### 2.9 DNA sequencing

The FSHR N680S variants were analyzed and confirmed with PCR amplification and Sanger sequencing using a previous protocol (23).

### 2.10 Statistical analysis

The clinical specificity, sensitivity, positive predictive value (PPV) and negative predictive value (NPV) were calculated in the final clinical assessment with 95% confidence interval. Sanger sequencing was set as reference when comparing the results. All statistical analyses were performed using Stata Statistical Software version 18.0 (StataCorp LLC, College Station, TX, USA).

## 3 Results

### 3.1 Allele-specific LAMP

Positive reactions were detectable within 30–45 min when running the LAMP analysis in gradient temperatures between 60-65°C. The shortest reaction time was observed when the temperature was set to 65°C, which was therefore applied in further analyses. By comparing the colorimetric discrimination of N and S variants, a primer set with each inner primer comprising only one mismatched nucleotide adjacent to the SNP revealed the most accurate discrimination.

**Figure 2:**
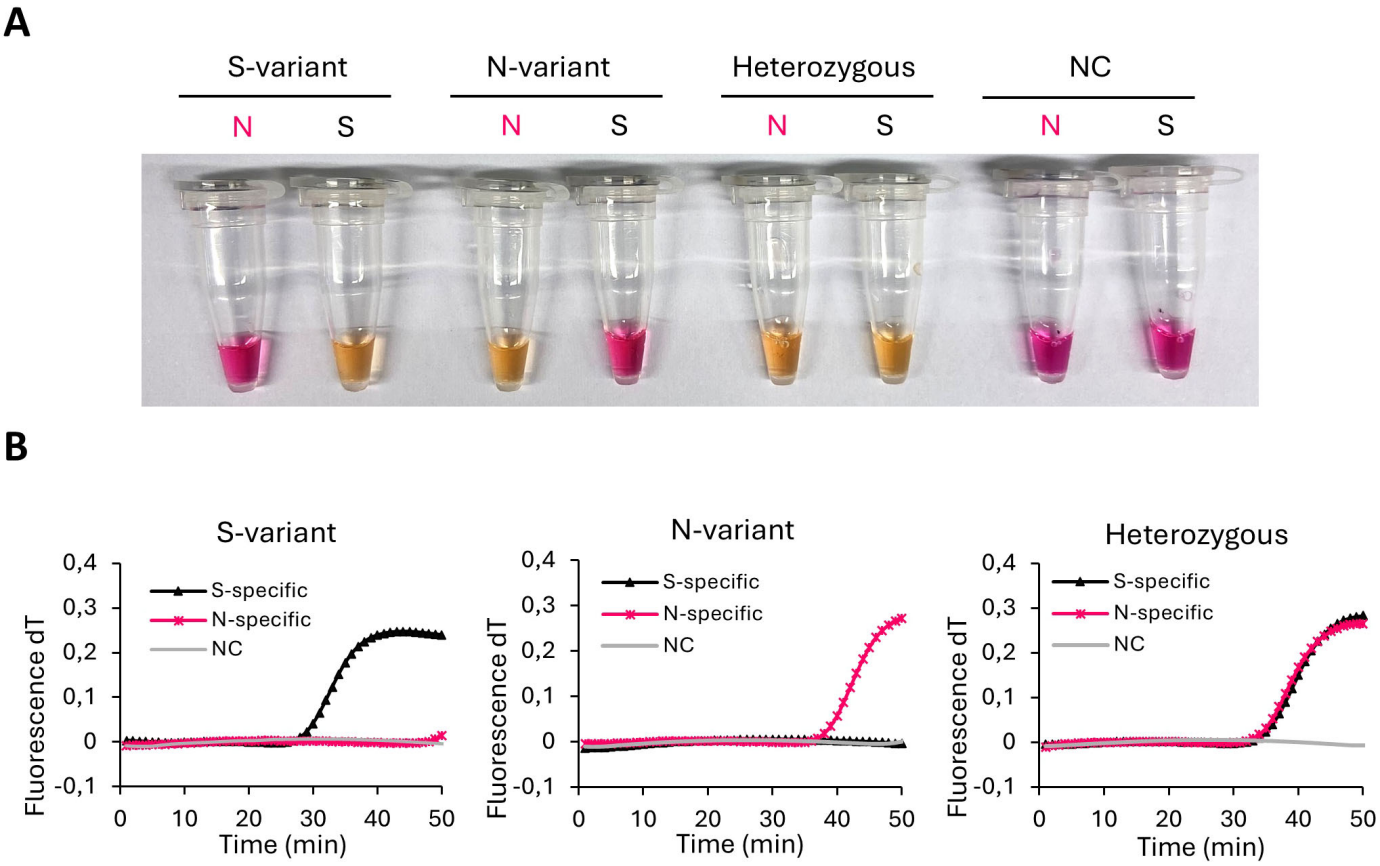
Genotyping of FSHR N680S. Representative image of LAMP genotyping of the FSHR N680S using **(A)** visual detection and **(B)**, fluorescent amplification curves performed with S-specific and N-specific primer sets using DNA templates of the variants homozygous SS, homozygous NN and heterozygous NS. S serine; N asparagine; NC negative control.

A color change from pink to bright yellow occurred in all samples containing matched primer sets and templates (**Figure 2A**). Reactions with unmatched templates remained pink. In reactions containing heterozygous DNA, a color change was observed in both assays. Correspondingly, in real-time PCR reactions with matched primer sets and templates, a rapid increase in fluorescence was observed within 50 min (**Figure 2B**). In contrast, no fluorescence was observed in reactions with unmatched templates. All results were confirmed by Sanger sequencing. Colorimetric results were consistent with fluorescent results.

### 3.2 Performance validation

**Figure 3:**
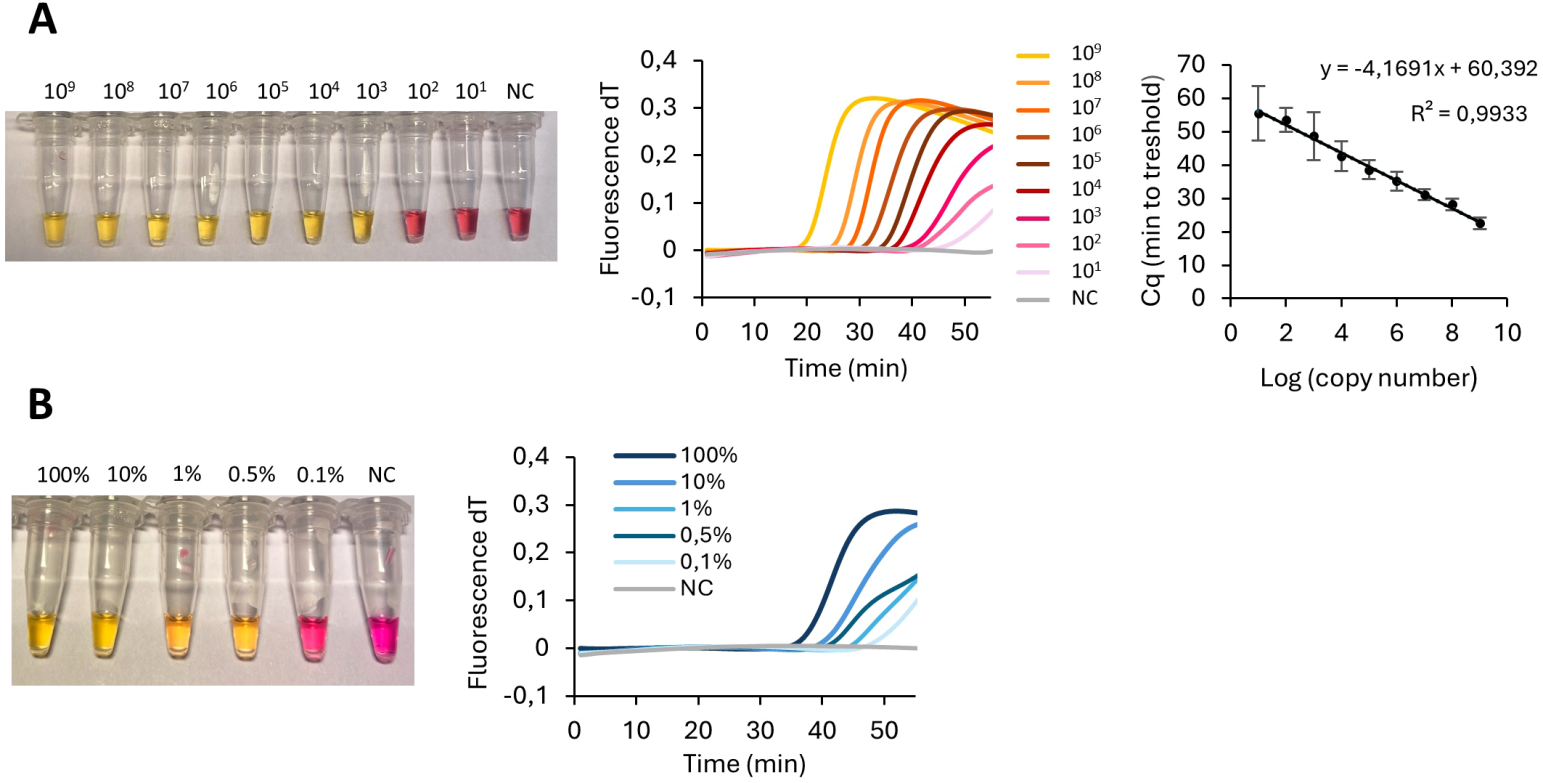
Sensitivity and specificity analysis. **(A)** Visual detection (left) and fluorescent amplification curve (right) of LAMP performed with S-specific primers with 10-fold dilutions of the S-allele containing plasmid. The linear relation between the Cq value and the logarithm of the copy number plasmid. **(B)** Visual detection (left) and fluorescent amplification curve (right) of LAMP performed with S-specific primer set with varying ratios of S-allele and N-allele containing plasmids (100, 10, 1, 0.5, 0.1%) in final concentration 10^5^ copies/µL. S serine; N asparagine; NC negative control.

The colorimetric sensitivity analysis suggested that the LAMP can successfully detect quantities of 10^3^ copies DNA template within 30 minutes (**Figure 3A**). Within 50 minutes, 100 copies of the target plasmid could efficiently amplify the sequence to reach fluorescence above the threshold level. The Cq values were linearly correlated with the target concentration. In DNA extracted from whole blood and crudely processed buccal swab samples, a concentration translatable between 10^4–^10^5^ copies was expected (20).

The specificity analysis revealed that the LAMP assay was able to detect the S variant in a proportion of <1% (**Figure 3B**). The addition of the PNA clamp in the colorimetric analysis resulted in more samples being genotyped correctly 95% (48/50 samples) compared to 35/50 (70%) in the absence of the PNA clamp.

**Figure 4:**
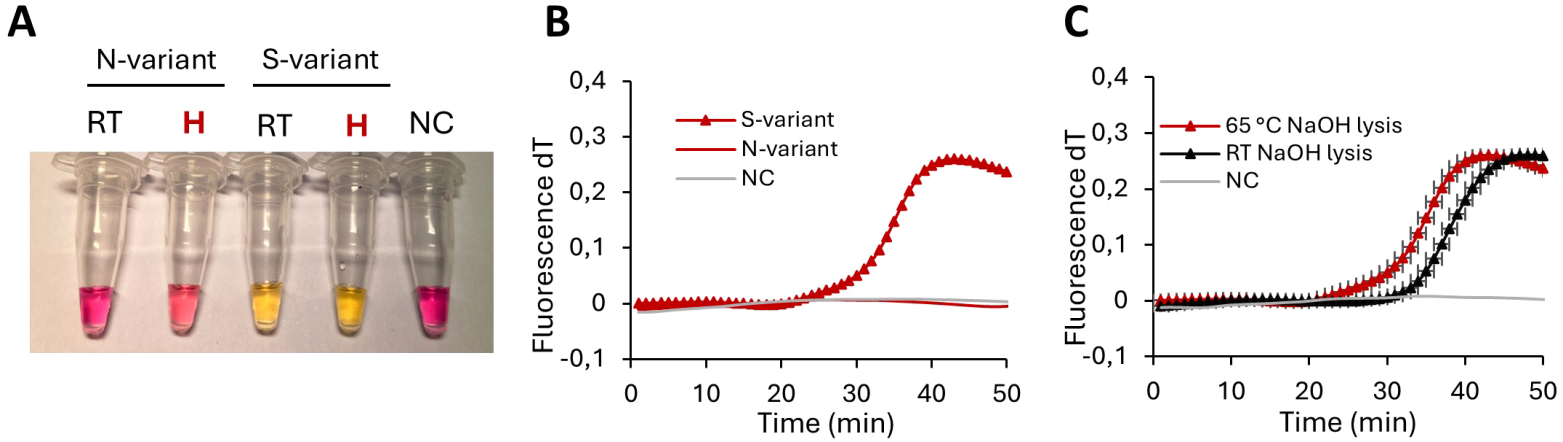
Crude sample analysis. Visual detection and fluorescent amplification curves of LAMP performed with S-specific primers. **(A)** Buccal SS template treated in NaOH lysis buffer at 65°C or room temperature. **(B)** Buccal samples homozygous SS or homozygous NN variants kept in NaOH lysis buffer at 65°C. **(C)** Homozygous SS or NS variants in lysis buffer at 65°C or room temperature. S serine; N asparagine; NC negative control; RT room temperature; H heated at 65°C.

Preparing the assay for processing crude buccal swabs, 25 mM NaOH of was found optimal for cell lysis. When investigating the ability to discriminate between the S and N-variants in an S-specific assay, a color change was observed in reactions containing any S-variant DNA but not in reactions containing NN-variant DNA (**Figure 4A**). The results were validated in the fluorescence assay (**Figure 4B**). Incubation at 65°C as compared to RT lysis increased the amplification speed in the subsequent LAMP reaction when the S-variants were analyzed with an S-specific primer set (**Figure 4C**).

### 3.3 Clinical samples

#### 3.3.1 Part 1

In 400 previously sequenced blood samples, 120 samples were homozygous for N, 87 samples were homozygous for S, whereas 193 samples were heterozygous NS. When analyzed LAMP, 269/280 (96%) were found consistent with SS/NS by Sanger sequencing, whilst 111/120 (93%) samples were found consistent with NN.

#### 3.3.2 Part 2

In the second part, when 100 buccal samples were compared to blood DNA from the same persons, 32 samples were homozygous for N, 20 samples were homozygous for S, and 48 samples were heterozygous for NS by Sanger sequencing. With the LAMP assay, 91/100 samples (91%) were genotyped correctly, of which 59/68 samples (87%) were detected correctly as homozygous SS or NS, and 32/32 samples (100%) were distinguished correctly as homozygous NN. Of the 9 incorrectly genotyped samples, 7 samples were heterozygous NS, and 2 samples were homozygous for S. The clinical performance demonstrated 86.8% sensitivity [95% Cl 76.4-93.8%] and 100% specificity [95% Cl 89.1-100%]. The PPV was 100% (95% CI: 93.9–100%), and the NPV was 78% (95% CI: 62.4–89.4%).

## 4 Discussion

The major result of current work is a LAMP assay that can be used in guidance of the type of FSH to use for women undergoing ovarian stimulation prior to IVF, egg donation, or egg freezing. Taking the N680S variant into account in the choice of FSH stimulant has demonstrated to increase the number of follicles (8) as well as live birth rate in a previous study (7). The current rapid genotyping method was developed to serve as a simple test that can be performed on-site, at IVF clinics globally. The results can be seen with the naked eye within an hour, and a personal recommendation regarding the type of hormone to treat with can be made, rather than having to use empirical criteria for decision of which type of hormone to prescribe.

A well-known challenge regarding LAMP genotyping is the risk of contamination and non-specific amplification (24). In current work, mismatched hybridization between primers and target DNA was initially noted. We addressed this issue by adding a PNA oligo designed to serve as blocking agent for unspecific amplification (25, 26). This strategy increased the specificity of the assay and resulted in almost complete elimination of non-specific amplification.

DNA extraction has been another drawback for a rapid genotyping method. While the LAMP assay has proved to be a time-efficient alternative to conventional techniques in the genotyping of several common SNPs (26–28), most LAMP protocols still rely on DNA extraction before analysis. We applied a protocol where crude samples were treated with NaOH in order to shorten time-to-result and sample preparation. By adjusting the temperature and NaOH concentration to optimize cell lysis, the entire analysis was improved and could be completed within an hour. Compared to Sanger sequencing, which typically requires two or more days from sampling to results, a one-hour genotyping assay can easily be incorporated into workflows that demand immediate results for continuous treatment.

Moreover, our LAMP assay does not require any expensive equipment. The isothermal reaction is easy to perform with a standard water bath, heating block or other temperature regulators that can be set to 65°C. All analyses on clinical buccal samples in the study were performed at an IVF clinic by laboratory technicians. Results were available within an hour from start to finish. We therefore strongly believe that the analysis process is clinically feasible. In the final hospital setting, 91% consistency with the golden standard DNA sequencing was reached, with a PPV of 100% and an NPV of 78%. The assay was most likely to misclassify heterozygous carriers as homozygous NN carriers. In fact, all incorrectly genotyped samples were identified as false negative results, likely due to small amount of DNA from the swab as a longer incubation time in NaOH prior to genotype reaction revealed higher DNA yield Even samples with very low DNA concentration amplified eventually. However, the DNA yield from the swab likely varies between individuals and the possibility of incorrect results must hence be considered in a clinical context. Consequently, false negative genotyping would result in rFSH prescription to S-carriers, yet no new harmful risks would be introduced compared to routine care. In worst case scenario, the chance of pregnancy and live birth would be comparable to those achieved with no genotyping. False positive tests would result in homozygous NN carriers being prescribed hMG. Meta analyses directly comparing rFSH and hMG stimulation have not been able to identify the superior stimulant in terms of IVF outcome nor risk of complications (29). Hence, in both scenarios, the protocol would not introduce any residual risks compared to normal IVF routine.

The limitation of this study is the small sample size and recruitment of patients at a single center. Moreover, LAMP genotyping is not fully optimal for correct detection of heterozygous samples. A significant strength is that the assay has already been applied in a clinical setting by laboratory technicians in several steps, which has allowed us to identify and address challenges and technical errors.

The next step would be to conduct a randomized controlled trial, with one group using the LAMP-test to guide hormone selection for ovarian stimulation, whereas the other group would follow standard clinical routine. This would allow for a direct assessment of the feasibility and the clinical value of the test in IVF treatment.

## 5 Future perspectives

Many studies have shown that the FSHR N680S affects receptor sensitivity and, thereby, the concentration of serum FSH (10, 30, 31). In the male reproductive system, FSH has a role in regulating spermatogenesis (32), and FSH therapy has been tried for treatment against poor sperm parameters (33, 34). Some evidence points in the direction that the effect could be FSHR N680S dependent. In one study, only men homozygous for the NN allele showed improved sperm quality after twelve weeks of rFSH therapy (35), so far hMG has not yet been evaluated. Stratified treatment of men, based on results from the LAMP assay may hence be clinically applicable also for men with low sperm concentration. Hence, studies regarding this matter are warranted.

## 6 Conclusion

We have developed a simple, non-invasive and cost-effective genotyping assay based on colorimetric detection that can be completed within one hour. The rapid turnaround time allows for results to guide treatment decisions during ovarian stimulation. From a clinical perspective, the assay can be performed directly on crudely processed buccal samples by laboratory staff. This approach may contribute to simplifying and advancing precision medicine in assisted reproductive treatment.

## Data Availability

The datasets presented in this study can be found in online repositories. The names of the repository/repositories and accession number(s) can be found below: https://doi.org/10.5878/sk15-xx62, SND ID: 2025-227.
